# Association study of 15q14 and 15q25 with high myopia in the Han Chinese population

**DOI:** 10.1186/1471-2156-15-51

**Published:** 2014-04-27

**Authors:** Yu Qiang, Wenjin Li, Qingzhong Wang, Kuanjun He, Zhiqiang Li, Jianhua Chen, Zhijian Song, Jia Qu, Xiangtian Zhou, Shengying Qin, Jiawei Shen, Zujia Wen, Jue Ji, Yongyong Shi

**Affiliations:** 1 Bio-X Institutes, Key Laboratory for the Genetics of Developmental and Neuropsychiatric Disorders (Ministry of Education), Shanghai Jiao Tong University, Shanghai 200030, P.R China; 2 Schizophrenia Program, Shanghai Mental Health Center, Shanghai Jiao Tong University School of Medicine, Shanghai 200030, P.R China; 3 Wenzhou Medical College, Wenzhou 325003, P.R China; 4 Shanghai Changning Mental Health Center, 299 Xiehe Road, Shanghai 200042, P.R China; 5 Institute of Neuropsychiatric Science and Systems Biological Medicine, Shanghai Jiao Tong University, Shanghai 200042, P.R China

**Keywords:** RASGRF1 gene, High myopia, Refractive errors, Single nucleotide polymorphism, Correlation Analysis

## Abstract

**Background:**

Refractive errors and high myopia are the most common ocular disorders, and both of them are leading causes of blindness in the world. Recently, genetic association studies in European and Japanese population identified that common genetic variations located in 15q14 and 15q25 were associated with high myopia. To validate whether the same variations conferred risk to high myopia in the Han Chinese population, we genotyped 1,461 individuals (940 controls and 521 cases samples) recruited of Han Chinese origin.

**Result:**

We found rs8027411 in 15q25 (P = 0.012 after correction, OR = 0.78) was significantly associated with high myopia but rs634990 in 15q14 (P = 0.54 after correction), OR = 0.88) was not.

**Conclusions:**

Our findings supported that 15q25 is a susceptibility locus for high myopia, and gene RASGRF1 was possible to play a role in the pathology of high myopia.

## Background

Myopia, the most common visual disorder in the world, defined as a spherical refractive error of people who see near objects more clearly than far away objects because the images are focused on the vitreous inside the eye rather than on the retina. There is a high prevalence in populations of Asian (40%–70%) and European (20%–42%) descent [1,2]. And, high myopia, with prevalence 1%–2% in the general population, refers to myopic eyes with very long axial lengths (26 mm) or a high degree of myopic refractive error (6D). High myopia is associated with increased risk of the development of sight-threatening eye diseases, such as glaucoma, macular hemorrhage, retinal detachment, visual impairment, and blindness.

Recently, the incidence of high myopia has been increasing worldwide, especially in the younger East Asian population. In a population of Japanese students 3 to 17 years old, the prevalence of myopia increased from 49.3 to 65.6% [3,4]. In other countries, the prevalence of myopia shows variable ratio (36.7–87.2% in a Chinese, 19.8–62.1% in a general Asian group, 5.2–40.5% in a Caucasian group aged 5–17 years, and 2.3–14.7% in Australian children aged from 4 to 12 years [5,6]. Different countries and regions show considerable variability of prevalence rates [7]. The etiology of refractive errors and myopia is complex, and it is not fully understood. However, it has been agreed that both the environmental factors, such as proximity to work, higher educational background, [8] urbanization, outdoor activity, etc., and genetic factors play a role in developing myopia [9]. Numerous cross-sectional studies suggest that genetic heritability might be as high as 80% [10]. Segregation analyses suggest the involvement of multiple genes rather than a single major gene effect [11]. Linkage studies have reported many candidate genes of high myopia, including MYP1 on Xq28, MYP2 on 18p, MYP3 on 12q, MYP4 on 7q, MYP5 on 17q, MYP6 on 22q12, MYP7 on 11p13, MYP8 on 3q26, MYP9 on 4q12, MYP10 on 8p23, MYP11 on 4q22-q27, MYP12 on 2q37.1, MYP13 on Xq23-q25, and MYP14 on 1p36 [6,12-14].

Now, genome-wide association study (GWAS) is widely used to reveal the susceptibility genes of many complex diseases. However, no GWAS on refractive error or myopia has previously been reported until Solouki *et al.*[11] and Hysi *et al*. carried out GWASs in European descent populations and identified loci at 15q14 and 15q25 to be associated with common myopia and refractive error [10].

In 2011, Hayasbi et al. [3] performed a study to validate whether variations in chromosome 15q14 and 15q25 are associated to refractive error and myopia in the Japanese population, and their results illustrated that rs524952 in 15q14 was associated with high myopia but SNPs in 15q25 were not. Another study of the Han Chinese population conducted by Shi *et al.*[1] found the most significant loci in 15q14 and 15q25 were not associated with high myopia. However, they did not obtain the replication experiment data genotyping the specific loci in their study by using 419 cases and 669 controls.

In this study, we specifically selected the most significant SNPs reported in 15q14 and 15q25 to be our targets. We genotyped 940 unrelated normal controls and 521 unrelated individuals with high myopia of Han Chinese origin by Taqman technology.

## Methods

### Ethics statement

The completion of the entire research design and procedures involved were submitted to the ethics committee of Bio-X center Shanghai Jiao Tong University and got approved. We declare that our research was in accordance with the Helsinki Declaration (http://www.wma.net/en/30publications/10policies/b3/). Before the study began, each participant was clearly explained about the procedure and purpose of the study and consent to participate to the research. All data were recorded anonymously and participants could withdraw their file if requested.

### Samples

Sample information was shown in Table 1. All samples were selected from the east China. 688 controls were randomly recruited from Shanghai city, and 252 controls and 521 cases were recruited from Zhejiang Province. All cases were HM patients with myopia of -6.00D or less suffering from fundus injury. All controls were free of myopia and fundus diseases. The mean age of cases (200 males and 321 females) was 36 ± 14.95, and the mean age of controls was 42.5 ± 13.3 and 31 ± 10.66 based on two batches of samples respectively.

**Table 1:** Characteristics of the study population

	**Patients**	**Controls**	
	High myopia*	WZMC*	SHHP*
Patients, n	521	252	688
Age in years, Mean ± SD	36 ± 14.95	42.5 ± 13.3	31 ± 10.66
Sex, n			
Male	200(38.4%)	139(55.1%)	385(60.0%)
Female	321(61.6%)	113(44.9%)	303(40.0%)
Axial length, mm ± SD			
Right eyes	29.39 ± 2.85	–	–
Left eyes	29.93 ± 11.95	–	–
Refraction of the phakic eyes, D ± SD			
Right eyes	-14.14 ± 6.28	–	–
Left eyes	-14.01 ± 6.68	–	–

*Patients are from School of Optometry and Ophthalmology and Eye Hospital Ophthalmic Clinic, Wenzhou Medical College, Zhejiang Province, China.

*WZMC are DNA samples randomly selected from Wenzhou Medical College, Zhejiang Province, China.

*SHHP are samples randomly selected from Shanghai the Han Chinese population database.

### DNA extraction

All DNA samples were extracted from peripheral whole blood of each subject using a Tiangen DNA extraction kit (Biotech, Beijing, China). Genomic DNA was diluted to working concentrations of 10 ng/ml for the genotyping step.

### Genotyping

Two SNPs (rs634990, rs8027411) were selected based on their specific presence in two previous GWASs. Genotyping was carried out by a commercially available assay using the Taqman method on two platform (BioMark™96.96 Genotyping array, Fludigm, South San Francisco, CA) (TaqMan SNP assay with the ABI PRISM 7700 system; Applied Biosystems, Foster City, CA). The mean call rate for all markers was 99%.

### Statistical analysis

For our, association analysis of the sample was conducted by SHEsis (http://analysis.bio-x.cn), including the calculation allele and genotype frequencies, Hardy-Weinberg equilibrium, and pairwise linkage disequilibrium. The significance level was set at α = .05. There was no significant deviation from the Hardy-Weinberg equilibrium in the controls (P ≥ .05). To avoid the false positive results, we performed Bonferroni correction for P values.

## Results

We genotyped two SNPs (rs634990 and rs8027411) in 940 controls and 521 cases in total. The characteristics of our sample set were listed in Table 1. Results were shown in Table 2. The distribution of the genotypes of those two SNPs was in HWE (P ≥ 0.05).

**Table 2:** Association between phenotype of high myopia and genetic variants at 15q14 and 15q25 in the Han Chinese Population

**Chr/Mb**	**SNP [Minor Allele]**	**Case MAF**	**Allele Frequency**		**OR**	**P value**	Adjusted P*	**Genotype Frequency**			**OR**			**H-W P**
15/35.0	rs634990[C]	0.483	C	T				CC	CT	TT	CC	CT	TT	
	HM cases		450(43.4%)	586(56.6%)	0.88(0.76-1.04)	0.27	0.54	102(19.7%)	246(47.5%)	170(32.8%)	0.82(0.63-1.07)	1.01(0.81-1.26)	1.15(0.91-1.45)	0.45
	Controls		858(46.6%)	984(53.4%)				212(23.0%)	434(47.1%)	275(29.9%)				0.11
15/79.5	rs8027411[G]	0.441	G	T				GG	GT	TT	GG	GT	TT	
	HM cases		396(38.7%)	626(61.3%)	0.78(0.66-0.91)	0.006	0.012	77(15.1%)	242(47.4%)	192(37.6%)	0.66(0.50-0.89)	0.99(0.80-1.23)	1.32(1.06-1.66)	0.96
	Controls		839(44.9%)	1029(55.1%)				197(21.1%)	445(47.6%)	292(31.3%)				0.26

Adjusted P* is the P value modified by Bonferroni’s mutable tests correction.

Finally, we found SNP rs8027411 in 15q25 was associated with high myopia (P = 0.012), and the risk allele was consistent with the previous report. However, another SNP rs634990 showed no association (P = 0.54, OR = 0.88[0.76-1.04]). According to the genotypic OR analysis, we observed an additive risk effect of allele T of rs8027411 (Table 2), which is consistent with the allelic result. There was no deviation from Hardy-Weinberg equilibrium in the control subjects of each SNP.

## Discussion

High myopia has been thought to be a complex disease which is affected by multiple factors, and many studies have revealed the susceptibility genes associated with high myopia. Several chromosome loci have been reported to be associated with common myopia, high myopia, or both. However, as we mentioned previously, the consistency among those reports is poor. In our study, we validated rs8027411 in 15q25 to be associated with high myopia identified in the Han Chinese population. The same SNP was reported recently in a GWAS of Caucasians, although the Caucasian cohort analysis was population-based and the proportion of patients with high myopia was very rare (1.7%–4.0%).

**Figure 1:**
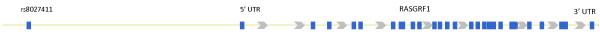
Integrated maps: genomic context and SNPs information of the rs8027411.

The validated SNP rs8027411 is on chromosome 15q25 and locates in the transcription initiation site of *RASGRF1* (Figure 1), it encodes Ras protein-specific guanine nucleotide-releasing factor1 [10]. The gene *RASGRF1* location and SNP rs8027411 information are shown in Figure 1. *RASGRF1* is a large gene which extents 130 kb, including 28 known exons and various mRNA transcripts (Figure 1). Hysi *et al.* found that *RASGRF1* expression can activate Ras by encoding protein which is highly expressed in neurons and retina in mice implicating this gene has functional influence on myopia pathogenesis [10]. Furthermore, the expression of *RASGRF1* is up-regulated by stimulated level of muscarinic receptors and retinoic acid [15,16]. Another evidence of knockdown model indicating that *RASGRF1* is contributed to myopia is that knockdown mouse models remain normal brain structure but have worse performance in exercises including long-term memory than wild-type mice [17]. It is caused by Lack of *RASGRF1* encoding, which causes severe deficiencies in photoreception and visual sensory processes though it remains a morphologically complete retina. More profoundly, it can alter downstream expression of many genes, including genes causing severe Mendelian vision disorders [17]. Above all, *RASGRF1* appears to be related in the maintenance of normal function of the retina and possibly in the signaling pathways determining myopia.

Comparatively, C. Klaver *et al.* identified a susceptibility locus rs634990 in 15q14 with a genome-wide association in a Dutch Population-based study [11]. Previous study showed that the gene *GJD2*, which is nearest to the susceptibility locus rs634990 and *RASGRF1* play important parts in the transmission and processing of visual signals which further continue or halt eye growth originated within the retina [18,19]. However, we found no significant association between rs634990 and high myopia, with P value = 0.54, OR = 0.88 (0.76-1.04). The limitation of sample size or genetic heterogeneity can be the possible reason.

Recently, Verhoeven *et al.* reported a comprehensive Genome-wide meta-analysis of multi-ancestry cohorts identified multiple new susceptibility loci for refractive error and myopia [20]. According their findings, rs524952 in 15q14 (P = 1.44 × 10^-15^) and rs4778879 (P = 4.25 × 10^-11^) in 15q25 showed genome-wide significant association with myopia. We noticed that rs524952 is adjacent to rs634990 with a distance between them less than 200 bp; and the distance between rs8027411 and rs4778879 is about 88 kb. By a linkage disequilibrium (LD) analysis based on HapMap CHB and JPT samples, we got pairwise D’ = 1, r^2^ = 1 for rs634990 and rs524952, and pairwise D’ = 0.75, r^2^ = 0.47 for rs8027411 and rs4778879. Our result of rs634990 (adjusted P = 0.54) indicated rs524952 was not associated with HM in the Han Chinese population, while the association of rs4778879 was considered to be supported by our result of rs634990 (adjusted P = 0.012).

Compare to the validation study performed in the Japanese population, we selected extremely high myopia patients to be cases, and all controls were free of myopia and fundus diseases. Moreover, our sample size is larger. However, independent studies with larger sample set will be more helpful. Pathological myopia is distinguished from common myopia or low/moderate myopia by excessive increase in axial length of the eyeball, which is the most important contributor to the myopic refraction. According to Nakanishi et al.’s study in 2009, pathological myopia patients should have axial length greater than 26.0 mm in both eyes, and this criterion is equivalent to “refractive errors greater than -6 D” [20]. Therefore, the HM patients in our study can be considered as pathological myopia patients.

## Conclusion

We found that SNP rs8027411 is significantly associated to high myopia in the Han Chinese population. The association of rs634990 in 15q14 was not validated. Moreover, *RASGRF1* gene in 15q25 is considered to be the susceptibility gene, and it is involved in learning, visual processing and muscarinic signaling pathways, all of which are considered to be correlated with myopia [10]. The identification of this locus in large Han Chinese sample set may give valid evidence to the research of the pathogenesis of high myopia.

## Competing interests

The authors declare that they have no competing interest.

## Authors’ contributions

YS conceived, designed the study, and supervised the study. YQ carried out the population-based genetic studies, participated in the genotyping and drafted the manuscript. WL and QW participated in the genotyping and data analysis. ZL, KH and JC performed the statistical analysis. JQ, XZ and SQ organized sample selecting part. ZS and JS participated in the manuscript drafting. ZW and JJ helped the data collection and data analysis. All authors read and approved the final manuscript.
